# Evaluating Environmental Ward Performance: Insights From Acoustic, Lighting, and Temperature Monitoring in a Palliative Care Unit

**DOI:** 10.1177/19375867261455034

**Published:** 2026-06-10

**Authors:** Emil E. Jonescu, Benjamin Farrell, Chamil Erik Ramanayaka, Lori Delaney, Edward Litton, Talia J. Uylaki, Gareth Watts, Sadaf Asad, Bella Brigham, Frances Bellemore, Davinia Seah

**Affiliations:** 1 95980 Edith Cowan University, Perth, WA, Australia; 2 Hames Sharley, Perth, WA, Australia; 3 Gabriels Hearne Farrell Pty Ltd, South Perth, WA, Australia; 4 School of Engineering and Technology, 6939 Central Queensland University, Brisbane, QLD, Australia; 5 School of Nursing, Midwifery, 7932 University of Southern Queensland, Ipswich, QLD, Australia; 6 School of Nursing, Midwifery and Social Work, University of Queensland, Brisbane, QLD, Australia; 7 Intensive Care Unit, 85075 Fiona Stanley Hospital, Murdoch, WA, Australia; 8 School of Medicine, UWA, Crawley, WA, Australia; 9 Intensive Care Unit, St John of God Hospital Subiaco, Subiaco, WA, Australia; 10 Independent Researcher, Adelaide, South Australia; 11 Palliative Care Department, 2205 Royal Prince Alfred Hospital, Camperdown, Australia; 12 St Vincent's Clinical School, University of New South Wales, Sydney, NSW, Australia; 13 2787 St Vincent’s Hospital Sydney, Sacred Heart Supportive and Palliative Care Service, Sydney, NSW, Australia; 14 Faculty of Medicine, University of Notre Dame Australia, Sydney, NSW, Australia

**Keywords:** environmental monitoring, palliative care, PCU, healthcare spatial performance, light, noise, sound, temperature, postoccupancy evaluation

## Abstract

The physical environment of inpatient wards plays a critical role in supporting rest and care delivery. In palliative care, environmental conditions such as sound, lighting, and temperature directly influence patient comfort, circadian rhythms, and staff performance. However, few studies have quantitatively assessed these factors in functioning palliative care units (PCUs).

**Objectives:**

To evaluate the spatial and environmental performance of an acute PCU through sensor-based monitoring of acoustic exposure, light levels, and temperature.

**Method:**

A postoccupancy evaluation approach was employed to assess conditions within inpatient rooms. Environmental loggers recorded data continuously at 1-min intervals across two 1-month periods. Parameters included sound levels (Lmax, Leq, Lmin in A-weighted decibels) to reflect perceived loudness, lighting (lux), and temperature (°C). Measurements were benchmarked against World Health Organization and Australian guidelines for sleep-supportive healthcare environments. Data were collected from both single- and multibed rooms in a metropolitan tertiary hospital PCU.

**Results:**

Sound levels frequently exceeded recommended thresholds. Nighttime averages reached 54 dB(A), while daytime LAeq exceeded 60 dB(A), with minimal day–night variation (<6 dB), indicating sustained exposure. Lighting data showed repeated nighttime spikes above 20 lux and insufficient daytime illumination for circadian regulation. Temperature exhibited minimal diurnal variation (<2°C), falling short of conditions known to support sleep.

**Conclusion:**

Environmental monitoring revealed persistent deviations from sleep-supportive conditions. These stressors likely impact both patient well-being and staff performance. Findings underscore the need for evidence-based design strategies and translational research that position the built environment as an active contributor to holistic care. In palliative contexts, architectural design should enable rather than simply contain clinical practice.

## Introduction

Healthcare design plays a critical role in shaping the experience of patients, families and staff within clinical environments. In end-of-life (EoL) care, this relationship is particularly pronounced. Palliative care units (PCUs) are emotionally and physically complex spaces where clinical performance must coexist with patient comfort, psychological support, and familial presence. The design and performance of environmental systems including acoustic sound levels, lighting conditions, and thermal regulation can therefore influence perceptions of safety, quality, and dignity (Jonescu et al., 2024, 2025b; McLaughlan & Richards, 2023). These systems, however, are constrained by infection control requirements that limit the use of sound-absorbing materials, while clinical equipment and procedural demands restrict spatial adaptability. Likewise, lighting and temperature settings are often optimized for clinical tasks rather than for supporting circadian regulation and sleep quality.

Although the importance of the built environment in healthcare is widely acknowledged, few studies have rigorously evaluated the spatial performance of PCUs using real-time environmental data. Existing research has focused on intensive care or surgical recovery settings, where environmental control has immediate physiological consequences (Blume et al., 2019; Delaney et al., 2015). In PCUs, the stakes are equally high, albeit more qualitative: sleep disruption, environmental discomfort, and sensory overload can diminish not only patient and family well-being but also the capacity for emotional connection and reflective care.

... *environmental discomfort, and sensory overload can diminish not only patient and family well-being but also the capacity for emotional connection and reflective care.*

Healthcare environments constitute complex sensory landscapes in which patients’ biological rhythms, psychological states, and therapeutic outcomes are shaped by ambient conditions (Ulrich, 1991; Ulrich et al., 2008). For palliative care patients who may be experiencing pain, cognitive decline, medication side effects, or emotional stress, environmental quality is central to comfort and perceived quality of care (McLaughlan & Richards, 2023; Sagha Zadeh et al., 2018).

Excessive sound levels, perceived as noise, remain one of the most frequently cited stressors in hospitals. Measured sound levels in many hospitals exceed recommended thresholds due to alarms, staff conversations, clinical interventions, patient vocalisations, equipment, and corridor traffic (Busch-Vishniac & Ryherd, 2023; Delaney et al., 2017; Xie et al., 2009). Excessive sound levels have been linked to sleep disruption, increased heart rate, cognitive impairment, and stress, particularly in older and terminally ill patients (Chen & Tang, 1989; McKinley et al., 2013). The World Health Organization (Berglund et al., 2010; World Health Organization (WHO), 1999) recommends maximum sound levels of 30 dB(A) in patient rooms at night; a level rarely achieved in practice (Delaney et al., 2017).

Lighting is another key component that influences circadian rhythms, hormone regulation, mood, and sleep quality. Bright daytime exposure supports circadian entrainment, whereas even low nighttime light can suppress melatonin, affecting sleep initiation and quality (Blume et al., 2019; Figueiro & Rea, 2014). Based on controlled studies of circadian-effective illumination, daytime light levels of roughly 1,000–2,000 lux at eye level are considered beneficial for maintaining circadian alignment, while nighttime exposure should ideally remain below 10 lux to minimize melatonin suppression (Figueiro & Rea, 2014).

Institutional routines and artificial lighting that conflict with natural daylight cycles or vary inconsistently due to operational routines may therefore misalign patients’ circadian cues.

Thermal comfort is another often-overlooked factor affecting sleep and comfort of patients. Hospital Heating, Ventilation, and Air Conditioning (HVAC) systems prioritize infection control and ventilation, but they often neglect perceived warmth or coolness, particularly for frail, immobile, or elderly patients (Okamoto-Mizuno & Mizuno, 2012). Evidence suggests that ambient temperatures between about 18 °C and 22 °C support sleep onset and comfort in controlled environments (Czeisler et al., 2005; Ormandy & Ezratty, 2012). However, appropriate diurnal variation remains critical (Czeisler et al., 2005), as thermal discomfort can exacerbate pain, disorientation, or agitation in sensitive populations (Ormandy & Ezratty, 2012, 2016).

Despite extensive research on individual environmental parameters in clinical outcomes, little work has concurrently examined acoustics, lighting, and thermal conditions in PCUs. Private investigations have tended to isolate single domains, such as environmental comfort and perceptions (McLaughlan & Richards, 2023; Sagha Zadeh et al., 2018) or EoL environmental experience (Deodhar et al., 2016; Fridh et al., 2009), or have focused on intensive care contexts where psychological monitoring is more common (Jonescu et al., 2024, 2025b). This limited body of evidence highlights the need for integrated environmental assessment in PCUs where fluctuations arise from architectural orientation, mechanical system performance and ward-level operational practices, which are factors rarely captured through snapshot evaluation or survey-based evaluations.

This study conducted at Sacred Heart Health Service—St Vincent's Hospital Sydney, an acute PCU in central Sydney, addresses these gaps by examining real-world environment performance using calibrated data loggers recording sound levels, light intensity, and temperature at 1-min intervals in occupied single- and multibed rooms over 2 month-long periods. These objective data streams provide an empirical basis for evaluating PCU performance against published benchmarks. Findings are used to assess how environmental conditions vary during routine care and how these variations relate to design quality. These results can inform design decisions, operational procedures, maintenance schedules, and clinical policies aimed at improving patient comfort and safety.

The dataset provides a baseline reference that could inform future remodeling or refurbishment of the same facility. When combined with data from other wards or institutions, it could also contribute to developing a broader comparative database to support predictive analyses of environmental performance (e.g., modeling expected threshold exceedance based on room orientation, occupancy, materiality, fixtures fittings and equipment, and operational patterns).

This article forms part of a broader multidisciplinary post-occupancy evaluation (POE) of the same PCU currently under review (Jonescu et al., 2025a). Several companion manuscripts address different disciplinary audiences, each analysing subsets of the shared dataset through distinct methodological and analytical lenses.

## Methodology

### Study Setting

The study was conducted at a metropolitan public teaching hospital in central Sydney. The acute PCU is located on Level 4 in a separate building from the main acute hospital, reflecting a model that distinguishes it from traditional inpatient hospice units (Mercadante & Bruera, 2023). The ward has dual aspects facing Darlinghurst Road and Victoria Street, which correspond to the two monitored room sets. There are no substantial external obstructions affecting daylight access on either façade. Street trees are positioned approximately 15–17 m from the building (near the street edge) and do not exceed the height of the fourth floor. The nearest neighboring buildings are roughly 40 m away on the other side of the abovementioned roads, one of comparable height and another of two storeys, neither of which produces measurable overshadowing. The remaining surrounds consist of manicured gardens and low vegetation, providing clear sky exposure.

The 14-bed unit has an average length of stay of 14 days. It is designated as a Level 5 Specialist Palliative Care Service, the highest classification under national guidelines, responsible for managing complex palliative care needs (Jung et al., 2022; Palliative Care Australia, 2018). The PCU building was constructed in 1987. Although the original design documentation was available for review, the spatial configuration suggests an intent to balance patient privacy, family accessibility, and staff visibility, consistent with contemporary principles of palliative care design. Although the study represents a POE, the unit had been in continuous operation for more than a decade at the time of monitoring, providing a mature environment for assessing long-term operational performance.

Two inpatient rooms were selected for monitoring: one single-occupancy room and one four-bed shared room. This enabled comparative analysis of environmental variation based on occupancy type (see Figure 1).

**Figure 1.**
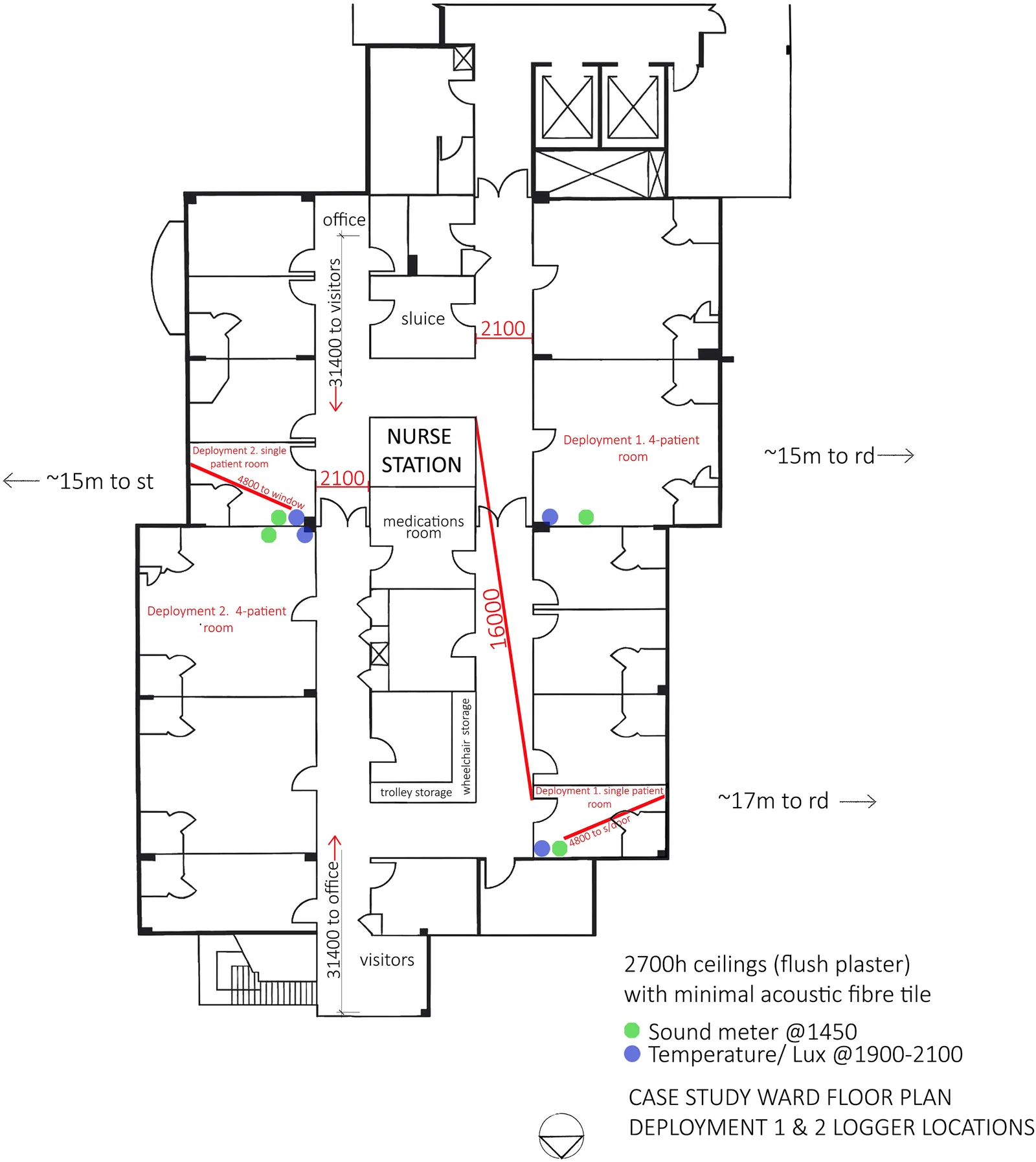
Palliative care unit ward floor plan showing the location of single- and multibed patient rooms selected for environmental monitoring, primary circulation routes, adjacent corridor zones, and the relative position of the monitored rooms within the overall ward layout. *Source.* Authors (2025). Plan of the single-occupancy room illustrating architectural layout, bed orientation, ensuite access, placement of data logging sensors for acoustic, lighting, and thermal monitoring, and distinguish internal corridor window and doors versus external glazing/sliding doors. Internal elevations of the same room showing ceiling height, window configuration, fixtures fittings and clinical equipment zones, and lightfittings influencing illuminance distribution. *Source*. Drawing adapted from Australasian Health Facility Guidelines (AusHFG, 2016), modified by the Authors (2025) based on on-site measurements and drawings taken during field investigation. Plan of the four-bed shared room highlighting bed arrangement, privacy curtain positions, and sensor locations for sound, light, and temperature monitoring, and distinguish internal corridor window and doors versus external fixed glazing. Internal elevations of the same room showing ceiling height, window configuration, fixtures fittings and clinical equipment zones, and light-fittings influencing illuminance distribution. *Source*. Drawing adapted from AusHFG (2016), modified by the authors (2025) based on on-site measurements and drawings taken during field investigation.

The single-occupancy room in the PCU measured 16.5 m^2^ (see Figures 1(a)–(b)), while the four-bed shared room was approximately 52 m^2^ (see Figures 1(c)–(d)). Each room was fitted with a 2.7 m flush plasterboard ceiling (noise reduction coefficient unknown), which included multiple panel openings around service penetrations such as ventilation outlets. Flooring was seamless vinyl throughout, and blue medical-grade privacy curtains were installed around each bed. Adjacent to each bed were melamine storage cupboards.

Each room featured an ensuite bathroom with walk-in access, separated by a timber-hinged door, along with a handwashing basin, full-height painted walls, coved vinyl skirting, overhead fixed-intensity LED downlights, and individual bedside spotlights. In both single and multibed rooms, televisions were ceiling-mounted at the foot of each bed. External glass sliding doors and internal corridor windows are both equipped with adjustable blinds. For the case study beds, the head of the bed in the single room was located approximately 1.5 m from the corridor window and 3.0 m from the external glazing. In the multibed room, these distances were approximately 1.5 m and 6.0 m, respectively (see Figures 1(a)–(d)).

Various clinical devices capable of producing intermittent sound were located at or above the bedhead area, including oxygen and suction outlets (with output depending on kPa settings), nurse call bells, wall-mounted telephones, and ceiling-mounted curtain tracks. Actual use of these elements varied between patient admissions.

The unit operates across several overlapping shift patterns: 07:00–15:30, 13:30–22:00, 07:00–19:30, 19:00–07:30, and 21:30–07:30. Clinical activity typically peaks between 08:00 and 10:00, although periods of heightened activity occur throughout both day and night depending on admissions, discharges, medical rounds, medication administration, personal care, allied health reviews, and the needs of patients and families requiring additional support. Visiting hours generally extend from 10:00 to 20:00, though flexibility is afforded to accommodate family and friends, including overnight stays when appropriate. Routine cleaning and maintenance activities are also undertaken within this period. Overnight operations are minimal, generally limited to medication rounds, comfort checks, and acute symptom management. These operational rhythms informed the definition of “daytime” and “nighttime” monitoring intervals adopted in this study.

### Data Collection

Daytime and nighttime periods were defined as 07:00–22:00 and 22:00–07:00, respectively. This segmentation was agreed by the research team in consultation with the nurse unit manager to align with on-site operations, including shift changeovers, ward rounds, visiting hours, and local daylight-saving hours during the monitoring months. Comparable POEs in critical care environments conducted in Australia have used similar monitoring windows to reflect clinical operations and light–dark cycles (e.g., Delaney et al., 2017). The adopted 07:00–22:00 window therefore captures the full span of diurnal clinical activity at this site while maintaining methodological consistency with common practice in ward-based environmental evaluations.

Environmental monitoring was conducted using precision datalogging equipment at 1-min intervals and subsequently aggregated to identify diurnal patterns and threshold exceedances. Detailed hourly plotting or reconstruction of daylight and electric lighting schedules was outside the scope of this study, which focused on evaluating overall environmental performance rather than system modeling. Acoustic conditions were monitored using a Convergence Instruments NSRT_mk4 sound level meter, fitted with a Type 1 microphone, and calibrated according to IEC 61672-1:2013 standards. Concurrently, lighting intensity and temperature were measured using HOBO^®^ MX2202 dataloggers, which provided integrated lux and thermal data at high temporal resolution. Lighting sensors were mounted facing upward to measure horizontal illuminance, capturing both direct and reflected light from overhead and lateral sources. Sensors were positioned near patient zones but out of direct luminaire beams to avoid glare and interference with clinical activities.

As most patients were bedbound, the risk of sensor obstruction in its location was negligible, ensuring measurements reflected typical patient light exposure under operational conditions. Photometric (illuminance) dataloggers were selected for their stability, accuracy, and suitability for continuous deployment in occupied clinical environments. While these devices do not capture full spectral power distribution, they provide reliable longitudinal measures of light intensity consistent with established healthcare POE protocols (e.g., Blume et al., 2019; Figueiro & Rea, 2014; Jonescu et al., 2025b).

Figure 1(a)–(d) illustrates the relative placement of sensors in relation to architectural features such as windows, bed orientation, and doorways. It also indicates adjacent corridor functions on the opposite side of the PCU, which, while not inherently “noise-generating” have potential to contribute to the acoustic environment depending on use (e.g., through movement of cleaning trolleys, IV stands, wheelchairs, Sara Steady devices, and rocker recliners).

Sensors were deployed across two 30-day periods: the first from 10:00 am on 5 December 2024 to 7:00 am on 5 January 2025 (Deployment 1), and the second from 10:00 am on 17 January 2025 to 7:00 am on 17 February 2025 (Deployment 2). Data collection periods were selected to capture multiple repetitions of typical diurnal (day–night) cycles and operational cycles reflecting weekday, weekend, and shift-based patterns of ward activity, rather than a single 24-hr observation. Sound exposure was assessed across the full 24-hr cycle to reflect not only potential for patient sleep disruption but also cumulative acoustic burden on staff.

Room selection included north-east and south-west orientations to account for differences in solar exposure and adjacency to external sound-generating sources. Equipment was positioned at bedside height to reflect actual patient environmental exposure, with placements confirmed in consultation with the ward's nurse unit manager to ensure clinical safety and unobtrusiveness.

#### Sound

Throughout this study, the term “sound” is used to reflect objective, instrument-based measurements. The term “noise” is only used in reference to published guidelines or measurement descriptors that employ this terminology (e.g., LAeq, WHO (1999) Community Noise Guidelines).

Sound level data were recorded as A-weighted Leq (equivalent continuous sound level), Lmax (maximum), and Lmin (minimum) at 1-min intervals. These metrics align with international guidelines for acoustic monitoring in healthcare, including those from the World Health Organization (Berglund et al., 2010), the Australian Standard AS 2107:2016, and the NSW Road Noise Policy. Sound levels were evaluated against established benchmarks, including a nighttime LAeq threshold of 30 dB(A) based on World Health Organization guidelines, and a maximum sound level (Lmax) threshold of 55 dB(A) derived from literature on sleep disturbance in healthcare environments (Jonescu et al., 2024). Recorded values were analysed against these benchmarks to determine the extent and frequency of exposure exceeding recommended thresholds for rest and sleep.

#### Temperature

Ambient temperature data were assessed using established optimal ranges for hospital comfort conditions, specifically between 20°C and 22°C with at least a 2°C diurnal variation preferred to support circadian temperature cycling and sleep physiology (de Dear et al., 2013; Okamoto-Mizuno & Mizuno, 2012). Excessively warm or cold periods were flagged for clinical concern.

#### Lighting

Lighting levels were assessed using photopic lux thresholds. This approach does not account for the spectral composition or biological effectiveness of light, as would be captured by melanopic equivalent daylight illuminance (mEDI; Brown et al., 2022) discussed in the limitations section. Separate thresholds were applied for daytime and nighttime analyses, with 1,000 lux used as a circadian target during daytime hours and >10 lux used to identify light disturbance during nighttime periods. Consistent with circadian lighting research, a target of approximately ≥1,000 lux for ≥30 min per day and mEDI literature (Lusczek & Knauert, 2021; Dewan et al., 2011; Duffy & Czeisler, 2009) we adopted a pragmatic indoors daytime target of 1,000–2,000 lux sustained for 30–60 min, using photopic illuminance as a practical proxy for circadian-effective exposure, and nighttime exposure less than 10 lux to minimize disruption (Blume et al., 2019; Figueiro et al., 2017). Temporal analysis allowed for evaluation of lighting consistency across shift periods and patient sleep windows.

### Data Analysis

Data were exported from the data loggers at 1-min intervals and screened for missing or anomalous values before analysis. To support temporal analysis, 1-min data were aggregated into hourly segments to visualize diurnal trends, while minute-level threshold exceedance events were tallied to identify short-duration cross-modal clustering. This enabled quantification of within-hour variation and the identification of time windows with frequent operational disturbances.

For each sensor location, minute-by-minute data were then aggregated to hourly means and maximums to examine diurnal trends and to daily means for overall comparison between rooms.

Summary statistics (mean, median, range, standard deviation) were calculated for each environmental parameter over the 2-month-long monitoring periods. Results were benchmarked against international performance thresholds. Data handling procedures and summary metric derivation followed the analytical framework previously applied in comparable intensive-care environmental monitoring studies (Jonescu et al., 2024, 2025b).

Data quality was assured through pre- and postcalibration of devices, secure transfer protocols, and manual inspection for anomalies or missing values. Sensor performance was verified through cross-checking colocated logger outputs and ensuring that readings were in alignment with expected operational ranges prior to analysis for both deployment phases. This methodological structure aligns with best practices in POE literature, particularly in healthcare environments where evidence-based design principles are applied (Andrade et al., 2017; Ulrich et al., 2004).

## Ethical Considerations

Ethical approval for the broader POE was obtained from the St Vincent's Health Network; St Vincent's Hospital Human Research Ethics Committee (HREC Approval #2024/ETH01551). This study employed a data-driven POE framework, a structured method used to assess how well a healthcare environment performs after construction and occupation. The overarching project investigated relationships between environmental conditions and patient or staff experience within the PCU. The present article reports only the objective environmental monitoring component of that study, focused on measured sound levels, light, and temperature conditions. No individual patient data were collected, and the monitoring devices recorded only environmental parameters without identifying information. All procedures complied with institutional and national research ethics standards for noninvasive environmental monitoring in clinical settings.

## Results

Continuous monitoring produced approximately 2 months of valid environmental data across single and multibed rooms, capturing typical ward operations and variation during both observation periods. During both monitoring periods, the unit remained near full capacity, consistent with its typical operational profile as a subacute palliative care service. The multibed rooms were continuously occupied, while the single rooms showed minimal turnover, with brief gaps of less than one day between admissions. Patient acuity was characteristic of palliative inpatients requiring complex symptom management and EoL care, with an estimated Nursing Hours per Patient Day of approximately six, typical for specialist palliative units in Australia. Staffing levels remained stable across both data collection periods, and no significant operational changes were reported.

### Acoustic Monitoring: Sound Levels Across Ward Spaces

Sound data collected across the two deployment periods revealed frequent exceedances of established acoustic thresholds in both the single-bed and shared (four-bed) rooms. Peak sound levels (Lmax) often surpassed the 55 dB(A) threshold, particularly during daytime operational hours (07:00–19:00). Across all monitored intervals, 62.4% of 1-min intervals in Deployment 1 and 64.1% in Deployment 2 exceeded this threshold equivalent to approximately 15 hr per 24-hr period in which sound levels were above recommended limits (see Figures 2 and 3). A full tabulated summary of LAeq, Lmax, and Lmin values by room type and deployment phase is provided in Supplemental Tables S1 and S2 (see Supplemental material), with corresponding trends illustrated in Figures 2 and 3.

*Across all monitored intervals, 62.4% of 1-min intervals in Deployment 1 and 64.1% in Deployment 2 exceeded this threshold equivalent to approximately 15 hr per 24-hr period in which sound levels were above recommended limits*.

**Figure 2.**
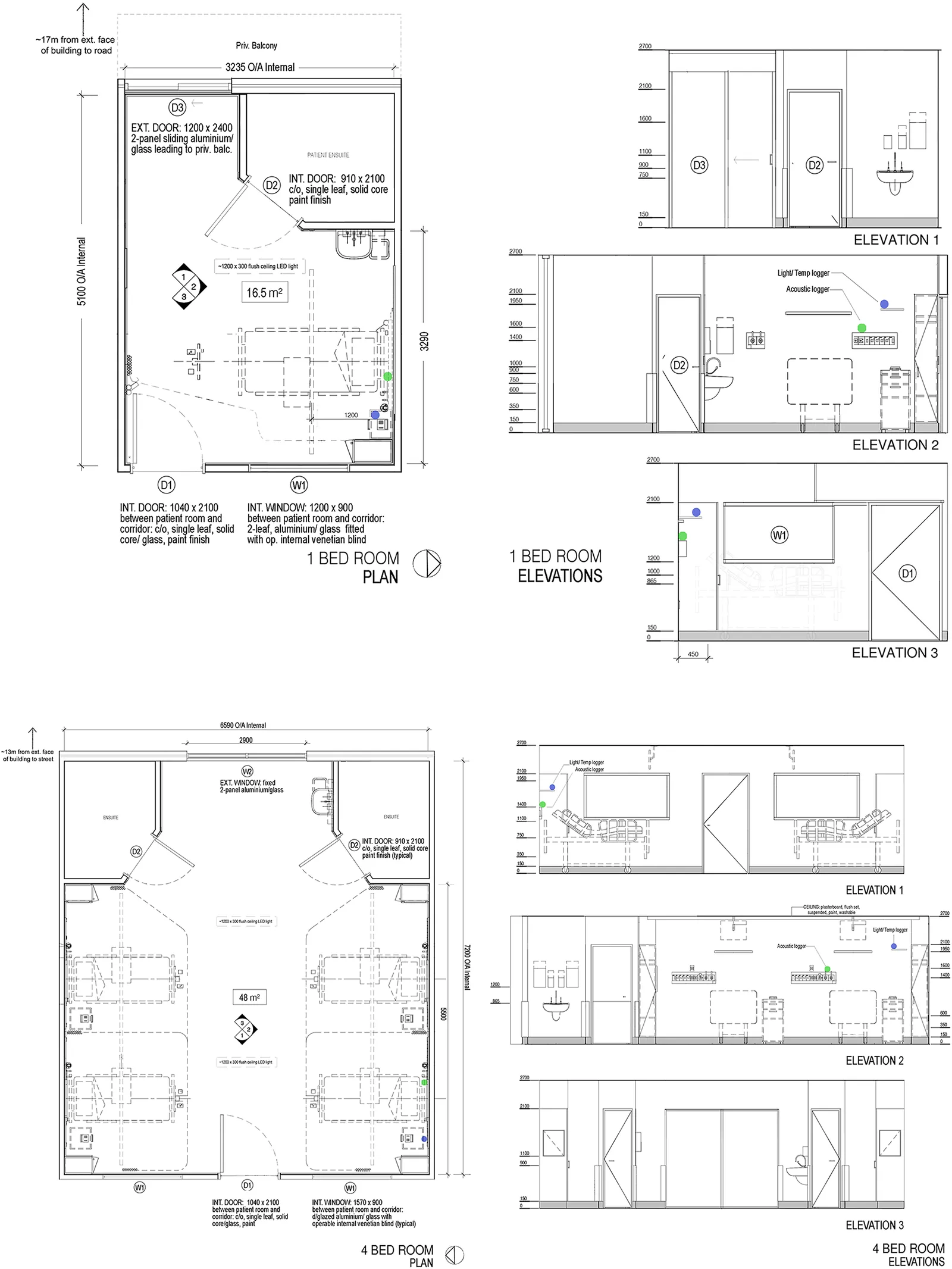
Plan of the single-occupancy room illustrating architectural layout, bed orientation, ensuite access, placement of data logging sensors for acoustic, lighting, and thermal monitoring, and distinguish internal corridor window and doors versus external glazing/sliding doors. Internal elevations of the same room showing ceiling height, window configuration, fixtures fittings and clinical equipment zones, and lightfittings influencing illuminance distribution. *Source.* Drawing adapted from Australasian Health Facility Guidelines (AusHFG, 2016), modified by the Authors (2025) based on on-site measurements and drawings taken during field investigation. Plan of the four-bed shared room highlighting bed arrangement, privacy curtain positions, and sensor locations for sound, light, and temperature monitoring, and distinguish internal corridor window and doors versus external fixed glazing. Internal elevations of the same room showing ceiling height, window configuration, fixtures fittings and clinical equipment zones, and light-fittings influencing illuminance distribution.

*Source.* Drawing adapted from AusHFG (2016), modified by the authors (2025) based on on-site measurements and drawings taken during field investigation.

**Figure 3.**
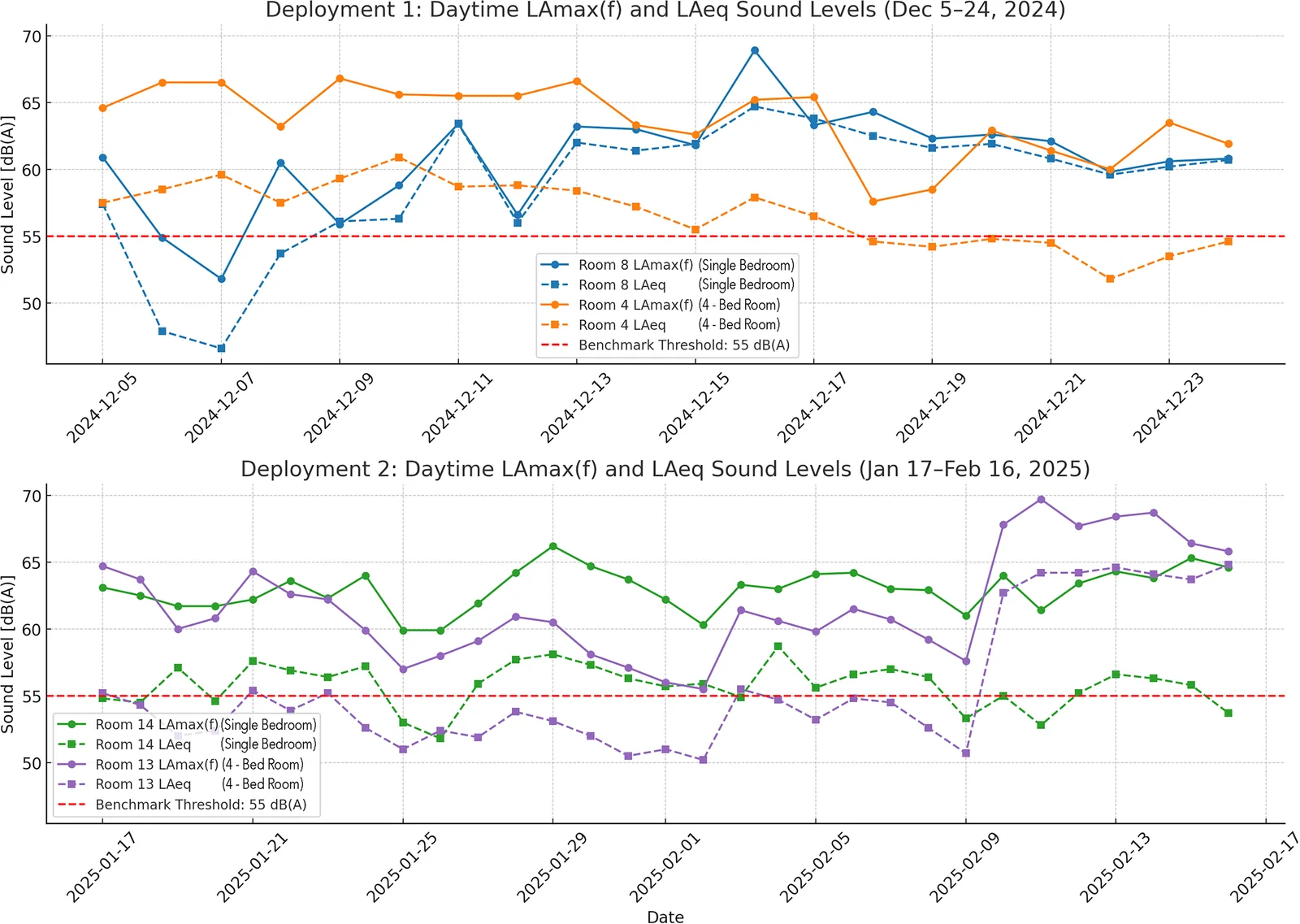
(Top) deployment 1—daytime lAmax(f) and lAeq sound levels (December 5–24, 2024) compares maximum and average sound levels in room 8 (single-bed) and room 4 (four-bed). Both rooms frequently exceed the 55 dB(A) threshold, particularly in LAmax(f), with four-bed rooms consistently showing higher levels. *Source.* Authors (2025). (Bottom) Deployment 2—daytime lAmax(f) and lAeq sound levels (Jan 17–Feb 16, 2025) shows room 14 (single-bed) and room 13 (four-bed) during the second deployment. Elevated and sustained LAeq levels, especially in the four-bed room, indicate ongoing acoustic burden above the recommended threshold.

*Source*. Authors (2025).

In the single-bed room, nighttime LAeq values exceeded the WHO-recommended 30 dB(A) limit for 72% of the monitoring period, equating to roughly 5 hr per night of exposure above the recommended level for nighttime hospital environments (Berglund et al., 2010). In the shared room, mean nighttime LAeq values exceeded 30 dB(A) for 74% of the monitoring period, compared with 72% in the single room (see Supplemental Table S1–S2).

Figures 2 and 3 illustrate daytime LAmax(f) and LAeq sound levels for each room type across both deployment phases, with a reference line at the 55 dB(A) threshold. Sound levels exceeded this threshold across a substantial portion of recorded intervals, particularly in multibed rooms. A full tabulated summary of LAeq, Lmax, and Lmin by room type and deployment is provided in Supplemental Tables S1 and S2 (Supplemental material).

### Lighting Performance: Day–Night Lux Consistency and Circadian Concerns

Analysis of lux data revealed suboptimal lighting conditions for circadian health. Daytime exposure (07:00–22:00) in both rooms showed inconsistent delivery of recommended light levels. Morning light levels (08:00–12:00) were below the 1,000 lux target. Across this period, between 2,082 and 2,729 1-min co-occurrence events were recorded per room (Table 3), representing approximately 15–20% of monitored intervals. In contrast, nighttime light exceedances (>10 lx) were infrequent, with low counts observed across all rooms during night and overnight periods (Table 3).

At night (22:00–07:00), several spikes exceeded 20 lux, double the accepted threshold for rest-oriented environments (<10 lux), affecting the circadian rhythm. Nighttime lux levels occasionally exceeded 20 lux, with short-duration spikes observed between 22:00 and 02:00 in both deployments. These events were more frequent in the shared room, consistent with proximity to corridor-facing openings. During the nighttime period (22:00–07:00), 3.3% of observations recorded ≥10 lux. Nighttime light levels in the single-bed room showed greater variability (*SD* = 30.9 lux) than in the shared room (*SD* = 3.3 lux). This variation corresponded to short-duration light level increases recorded between occupancy periods.

### Temperature Regulation: Static Conditions and Lack of Diurnal Variation

Thermal data captured a consistently narrow range of temperature variation across both rooms, with average daily variation <1.5°C. This flat diurnal profile deviates from optimal sleep physiology, where a minimum 2°C variation is associated with circadian signaling and sleep induction (Okamoto-Mizuno & Mizuno, 2012).

Room temperatures remained largely within the 20–24°C band, which meets general comfort criteria but may not fully support optimal sleep-related thermoregulation for older or critically ill patients. Notably, temperatures in the shared room tended to be warmer, see Tables 1 and 2 for summary of mean, maximum, and minimum temperature levels by time segment, deployment, and room type.

**Table 1.** Temperature Deployment 1 (Dec 5–24, 2024) and Deployment 2 (Jan 17–Feb 16, 2025). *Source*. Authors (2025).

Room Type	Deployment	Mean Temp (°C)	Day Mean (°C)	Night Mean (°C)	Diurnal Diff (°C)	Max Temp (°C)	Min Temp (°C)	Time Above 22°C (mins)
Single-bed room	1	21.08	23.98	22.11	1.87	25.22	20.12	1,871 (∼1.3 days)
Multi-bed room	1	21.41	23.74	22.04	1.7	25.22	20.25	4,653 (∼3.2 days)
Single-bed room	2	21.38	22.74	21.09	1.65	23.25	20.76	2,291 (∼1.6 days)
Multi-bed room	2	21.79	22.68	21.12	1.56	23.29	20.68	13,942 (∼9.7 days)

**Table 2.** Light Deployment 1 (Dec 5–24, 2024) and Deployment 2 (Jan 17–Feb 16, 2025). *Source*. Authors (2025).

Room Type	Deployment	Mean Light (lux)	Max Light (lux)	Time Above 10 Lx (Night) (mins)	Time in Optimal Day Range (1000–2000 Lux) (mins)
Single-bed room	1	69.66	750.08	4,366 (∼72 hr)	0
Multibed room	1	44.53	430.08	752 (∼13 (hr)	0
Single-bed room	2	25.99	370.4	546 (∼9.1 hr)	0
Multibed room	2	23.23	344.8	701 (∼11.7 hr)	0

### Cross-Modal Interference and Design Implications

When considered together, an analysis across the four monitored rooms revealed that environmental threshold breaches did not occur randomly but followed consistent, time-related operational patterns. Hourly peak count analysis revealed temporal clustering of noise events corresponding with known clinical shift overlaps. Table 3 presents the total number of noise peaks per period across four monitored rooms, aligned with operational transitions between day, evening, and night shifts; Table 4 highlights exceedance rates.

**Table 3.** Summary Table of Light and Sound co-Occurrences by Time of day Across all Rooms.

Time Period	Shift Overlap	4-Bed Darlinghurst Rd	4-Bed Victoria St	Single Darlinghurst Rd	Single Victoria St
Early morning (5:00–8:00)	Night-to-day shift overlap	1,064	549	616	513
Morning (8:00–12:00)	Morning and 12-hr day shift	2,270	2,137	2,082	2,729
Afternoon (12:00–15:00)	Morning + afternoon shift overlap	1,732	1,566	707	1,861
Late afternoon (15:00–18:00)	Afternoon and 12-hr day shift	1,949	1,303	827	1,046
Evening (18:00–21:00)	Evening + start of night shifts (19:00–21:30)	449	527	155	289
Night (21:00–00:00)	Full night shift period	2	37	0	7
Overnight (00:00–05:00)	Full night shift period	10	34	6	12

*Note.* Exceedance defined relative to context-specific thresholds: light >10 lux during nighttime periods and sound thresholds as defined in methods. Daytime light levels relative to the 1,000 lux circadian target are reported separately.

**Table 4.** Noise Exceedance Rates by Operational Period and Handover Window.

Period	LA(F)max Exceedance Rate	LAeq Exceedance Rate	Summary Interpretation	Typical Clinical Operations During This Time Window*
AM Handover (06:00–07:30)	70.0%	70.0%	Highest exceedance rates likely due to prep, verbal reports, equipment use.	Consistently showed elevated noise levels across multiple rooms. LAeq and LA(F)max exceedance rates were highest in this period for the majority of sites, implicating clinical handover activity, verbal reporting, and staff movement at the conclusion of overnight shifts.
Midday handover (13:00–15:30)	70–85%	70.8%	Sustained overlap of 2 shifts; noise from activity, visitors, meals.	Exhibited the highest rate of LAeq exceedances, often reaching or exceeding 70–85% in four-bed rooms. This suggests sustained background noise likely associated with overlapping shift presence, increased clinical throughput, meal rounds, and visitor-related activity.
PM handover (19:00–21:30)	35.0%	31.9%	Significantly lower exceedance suggests quieter transition to night.	Demonstrated lower LAFmax and LAeq exceedance rates relative to both morning and midday handovers. In several rooms, evening transition periods recorded lower noise than even non-handover intervals, suggesting possible behavioral or procedural adaptations to support patient rest.
Nonhandover times	45–65%	47.6%	Baseline rate still high but lower than morning and midday handovers.	Despite having the largest share of total readings, exhibited moderate exceedance rates (typically 45–65% for LAFmax), indicating a persistent underlying acoustic load even outside of structured transition periods.

*Note. Based on standard ward routines, not directly observed.

The morning period (08:00–12:00) recorded the highest peak noise activity across all rooms, with 2,729 peaks in the Single Victoria St room, 2,270 in the four-bed Darlinghurst Road room, and over 2,000 in both remaining rooms. This period coincides with the convergence of the morning (07:00–15:30) and 12-hr day shift (07:00–19:30).

The afternoon period (12:00–15:00), which includes overlap between morning and afternoon shifts (13:30–22:00), also exhibited substantial peak counts, particularly in the Single Victoria St room (1,861) and four-bed Darlinghurst Road room (1,732).

A decline in noise activity was observed during the late afternoon (15:00–18:00) and evening (18:00–21:00) periods across all rooms, although noise peaks remained present—especially in multibed configurations.

The night period (21:00–00:00) and overnight window (00:00–05:00) showed the lowest frequency of noise peaks across all rooms, with counts approaching zero in some single-bed rooms. This corresponds to the lowest observed noise peak counts across all rooms.

Illuminance monitoring showed that daytime light levels (07:00–22:00) were consistently below 1,000 lux, yielding a 100% daytime “subthreshold” occurrence against the specified criterion (<1,000 lux). This threshold (1,000 lux) represents a daytime circadian lighting target and is distinct from the nighttime light exceedance threshold (>10 lux) used in cross-modal and disturbance analyses. Across the same daytime window, noise exceedance was also elevated, with peak acoustic activity occurring during periods of persistently low daytime illuminance.

Nighttime light exceedances (>10 lx) were not consistently accompanied by concurrent acoustic spikes. In some instances, light exceedances occurred without corresponding increases in sound levels, while in others, short-duration acoustic spikes were temporally aligned with increases in light levels, particularly in shared rooms. These co-occurrences were most frequently observed in rooms on the Darlinghurst-facing side. These patterns were not uniformly present across all rooms or time intervals.

Conversely, acoustic exceedances (>30 dB(A) at night; >55 dB(A) Lmax during daytime periods) were sporadic, localized, and rarely cotemporal across rooms. Their lack of co-occurrence does not support a dominant, uniform building-services driver across rooms.

These cross-modal patterns are further interpreted in the Discussion in relation to their operational origins and design implications.

## Discussion

This study presents a POE of a PCU, incorporating continuous monitoring of three environmental variables: acoustic levels, light intensity, and ambient temperature. Accordingly, the study's contribution is methodological transferability and benchmarked exposure characterization, rather than statistical generalization or the establishment of universal PCU norms. These are known to affect patient well-being and sleep quality. The analysis offers empirical insights into how the ward performs relative to environmental best-practice benchmarks. The findings underscore the critical role of environmental quality in EoL care, revealing persistent exceedances of sound thresholds, insufficient circadian-supportive lighting, and inadequate diurnal thermal variation.

While few studies have quantitatively assessed environmental performance in PCUs, related investigations provide valuable context for interpreting these findings. McLaughlan and Richards (2023) observed that perceptions of comfort and homeliness in palliative environments were closely tied to lighting and acoustic privacy, an observation reflected here through inconsistent lighting levels and frequent sound exceedances. Sagha Zadeh et al. (2018) similarly identified that environmental stressors such as glare, thermal discomfort, and noise can diminish the quality of life and symptom management for palliative patients. Studies in critical care contexts (Jonescu et al., 2024, 2025b) have demonstrated that intensive care environments also struggle to meet acoustic and circadian lighting benchmarks, further suggesting that sensory imbalance is a systemic issue across healthcare settings. Collectively, these findings reinforce that while PCUs differ in operational intensity from ICUs, they remain subject to similar environmental challenges that can compromise rest, communication, and dignity at the end of life.

Sound levels across the ward exceeded the World Health Organization's recommended nighttime threshold (Lmax >30 dB(A)) as well as the acoustic criteria specified in Australian Standard 2107:2016 limits, particularly in multi-occupancy rooms. Common peak contributors were likely human-generated transient sounds such as conversation, alarms, and equipment movement (Jonescu et al., 2024; Naef et al., 2022). These exceedances coincided with periods of peak clinical activity, suggesting that ward operations are not acoustically buffered from patient areas. This is particularly problematic in PCUs, where calm environments support dignity, communication, and emotional stability for terminally ill patients (Busch-Vishniac & Ryherd, 2023; Xie et al., 2009). The data aligns with findings from ICUs and oncology wards, where elevated sound exposure correlates with disrupted sleep, increased stress biomarkers, and circadian desynchrony (Delaney et al., 2017; Jonescu et al., 2024; Litton et al., 2016), and the prevention and treatment of delirium, a highly prevalent condition in patients who are at the end of life (Watt et al., 2019).

Lighting levels revealed poor alignment with circadian principles. The temporal pattern of light exposure further supports this interpretation. Daytime light levels remained below 1,000 lux for 100% of recorded intervals, while the same period was marked by peak noise exceedance. This co-occurrence indicates that patients were routinely subjected to acoustically overstimulating but visually understimulating environments; conditions that are misaligned with circadian support and rest activity balance. Daytime illuminance frequently fell below the 1,000–2,000 lux range needed for circadian entrainment, while nighttime levels often exceeded the 10 lux threshold considered disruptive to melatonin production (Blume et al., 2019; Figueiro & Rea, 2014). Given that lighting interventions are among the most cost-effective and noninvasive strategies for improving sleep and mood in palliative care, this represents a missed clinical opportunity. Inadequate lighting is not merely a design flaw. It has direct implications for symptom management and quality of life (Çelik & Usta Yeşilbalkan, 2022).

Ambient temperature was consistently maintained between 20°C and 24°C but lacked diurnal variation. Warmer temperatures were recorded in shared rooms, likely due to greater occupancy, reduced ventilation flow, or heat-generating equipment. The literature suggests that dynamic temperature profiles, especially cooler conditions at night, support sleep onset and circadian rhythm (Czeisler & Buxton, 2005; Okamoto-Mizuno & Mizuno, 2012). For patients with compromised thermoregulation, such as those nearing EoL, fixed thermal conditions may increase discomfort or distress. These findings echo prior research in thermally sensitive settings such as aged care and critical care, where small adjustments in the thermal environment improved perceived comfort and sleep quality (de Dear et al., 2013; Simsek et al., 2014).

Although conditions met nominal compliance, overall performance fell short of standards conducive to clinical excellence in palliative care. Crucially, the findings highlight the potential for the built environment to influence clinical outcomes and patient experience, underscoring its role as an active determinant of environmental quality within care delivery. Indeed, the Australian Commission on Safety and Quality in Health Care have recommended that health service organizations should have policies and procedures that support “environmental care strategies, such as reducing noise … wherever possible for patients at risk of delirium or with delirium” (Australian Commission on Safety and Quality in Health Care, 2021).

### Environmental Tensions Between Clinical Requirements and Patient Well-Being

A key insight from this study is the need to navigate environmental tensions that exist between clinical requirements and patient well-being. Sound, light, and temperature each serve dual roles. They act as clinical enablers and, at the same time, potential sources of discomfort. These tensions should be explicitly addressed in both design and operational planning.

Lighting exemplifies this dual function. Clinicians require adequate illumination for tasks such as administering medication and observing patient symptoms, especially under low ambient light. However, intense or poorly timed lighting can disrupt patient sleep and circadian rhythms, which are already fragile due to illness and institutional routines. Effective lighting strategies require integrating task-specific illumination with circadian-sensitive illumination. For example, low, warm-spectrum lighting at night and bright, blue-enriched lighting during the day (Blume et al., 2019; Figueiro & Rea, 2014).

Thermal conditions also involve competing needs. Clinical staff require thermal stability to perform complex tasks effectively. Patients, many of whom are bedbound or metabolically vulnerable, may benefit from slightly cooler nighttime temperatures that support thermoregulation and sleep. Flexible, room-specific climate controls are essential to mediate these needs without undermining energy performance or staff comfort. It is also important to recognize that thermal preference varies considerably among patients, particularly older adults, who may experience altered thermoregulation. As such, some may prefer slightly warmer conditions than those typically considered optimal for sleep. Future design and operational strategies should therefore allow for localized individual thermal preference, enabling staff to adjust room conditions within clinically safe limits.

These tensions reveal that environmental factors are not merely design challenges. They are active clinical variables requiring ongoing negotiation across disciplines. Their management demands an integrated approach that considers simultaneous operational, architectural, and behavioral influences. Not all identified environmental burdens are solely attributable to architectural or building-services design. A proportion of observed disturbances, particularly those associated with temporal clustering of sound and light exceedances, are likely influenced by routine clinical activities such as staff movement, care delivery, and nighttime interventions. Accordingly, effective mitigation requires a dual approach: environmental design strategies (e.g., material selection, lighting control, spatial zoning) must be complemented by operational and behavioral considerations, including staff practices, workflow timing, and clinical protocols. Addressing environmental performance in palliative care settings therefore necessitates coordinated design and operational responses rather than reliance on spatial interventions alone.

*These tensions reveal that environmental factors are not merely design challenges. They are active clinical variables requiring ongoing negotiation across disciplines.*

### Operational Origins of Cross-Modal Interference

In practical terms, the act of illumination during clinical rounds involves low-level noise generation through door movement, footsteps, and short verbal exchanges—suggesting ongoing ambient and staff-related activity even after peak clinical hours. Short acoustic peaks coinciding with light spikes further indicate that these events are operational in nature, and their occurrence across multiple rooms reinforces this interpretation. This suggests recurrent activities such as staff rounds, caregiving, interventions, or corridor light spill during nighttime progression.

Short acoustic peaks coinciding with light spikes further indicate that clinical operational factors rather than environmental systems drive most multivariable environmental clustering. These overlapping stimuli reflect environmental conditions that are not optimally aligned with rest-orientated design objectives.

The findings reveal cross-modal interference patterns that highlight key opportunities for design intervention, where lighting intended for safety or visibility inadvertently intrudes on areas that impact patients. The spatial and temporal co-occurrence of acoustic and minor lighting events suggests that operational control dictates nighttime environmental performance, not the physical infrastructure. To help mitigate this, architectural strategies should therefore prioritize: (a) Enhanced nighttime light control and shielding strategies; (b) Spatial separation of care progression pathways; (c) Zoned corridors and room-level shielding controls to reduce light spill; and (d) Smart HVAC zoning to accommodate diurnal thermal cycling.

This operational explanation is reinforced by short-interval data showing that peaks in noise and minor light exceedance consistently occurred during clinical overlap periods. Specifically, the 08:00–12:00 and 13:00–15:30 windows exhibited both elevated acoustic activity and concurrent lighting deficits, suggesting that clinical workflows drive multimodal environmental intrusion. Across the 08:00–12:00 period, 37% of minutes exceeded the acoustic threshold, while 29% showed concurrent or temporally adjacent light exceedances. This adds empirical support to the notion of cross-modal impacts and validates its operational rather than incidental origin.

### Design, Policy, and Future Implementation

These implications are offered as intervention targets for similar contexts and should be tested and refined through harmonized multisite monitoring before being treated as normative guidance. From a design and policy standpoint, these findings support targeted retrofitting of environmental systems to better accommodate both patient rest and staff workflows. Acoustic dampening strategies such as surface treatments, alarm rationalization, and staff workflow considerations should be prioritized, particularly in multibed rooms. Lighting upgrades should aim for dynamic, tunable systems capable of diurnal modulation in both intensity and spectral quality. Where feasible, temperature systems should support 24-hr variability and allow patient or staff-controlled overrides where clinically appropriate.

Continuous monitoring offers a path forward. Embedding dataloggers or real-time dashboards into PCUs would enable proactive environmental management and allow staff to respond before thresholds are breached. This aligns with principles of evidence-based design and is increasingly viable given the affordability and miniaturization of sensing technologies (Ulrich et al., 2004).

This study shows that sensor-based POE is not merely a design evaluation method, but a clinical quality improvement tool. In PCUs, where patient sleep and dignity are deeply intertwined with environmental stability, environmental data should be treated as clinical data. Integrating it into design guidelines, training protocols, and facilities’ management practices would support more holistic and evidence-informed palliative care.

Current environmental recommendations for healthcare settings appear to be largely derived from acute or general ward contexts and may not adequately reflect the physiological and psychosocial needs of palliative patients. The findings of this study suggest that thresholds for sound, light, and temperature may warrant refinement to better align with the sensitivities and circadian vulnerabilities of hospitalized and EoL populations. For example, noise limits that are acceptable in acute wards may still disrupt sleep or communication for patients in prolonged or terminal care. Similarly, lighting requirements optimized for clinical tasks may not support rest in this group. Developing population-specific environmental benchmarks would require multisite studies that integrate objective monitoring with patient-reported outcomes, a direction that this work helps to inform.

### Limitations and Future Directions

Several limitations should be acknowledged. This study focused on a single acute PCU, limiting generalizability to other settings with different layouts, systems, or workflows. The findings should therefore be interpreted as site-specific exposure evidence and a transferable POE method, not as statistically representative estimates or normalized benchmarks for all PCUs. The monitoring period captured only two occupancy cycles across two room types, possibly omitting seasonal or intermittent variations. This limited temporal scope means that environmental parameters, particularly light exposure and ambient temperature, may not fully reflect the range of conditions experienced across different seasons. For instance, daylight intensity and duration vary significantly between summer and winter months, and external temperature shifts could influence HVAC operation and indoor comfort. In addition, key thermal comfort variables were not measured, including relative humidity, air speed, and mean radiant temperature. As a result, full comfort indices (e.g., PMV/PPD) could not be derived, and interpretation of thermal experience is necessarily limited to ambient air temperature patterns. Further, HVAC operational metadata (e.g., supply air temperature, airflow rates, diffuser/return operation, control setpoints, and scheduling/BMS logs) were not collected or accessible for this study, which prevents formal correlation testing between measured indoor conditions and HVAC system behavior. Future work should integrate multiparameter comfort sensing with concurrent HVAC operational data to enable attribution and to inform targeted design and commissioning interventions. Conditions may also differ markedly in stand-alone PCUs or hospices, particularly those embedded in garden or rural environments with reduced external stimuli. Future research should prioritize harmonized multisite comparative monitoring to enable pooled datasets and comparative reference ranges across PCU typologies. Such databases should pair environmental data with contextual descriptors (e.g., room type, occupancy, façade orientation, workflow intensity, and building-services characteristics) to support stronger cross-site interpretation and design guidance.

*In addition, key thermal comfort variables were not measured, including relative humidity, air speed, and mean radiant temperature.*

*Further, HVAC operational metadata (e.g., supply air temperature, airflow rates, diffuser/return operation, control setpoints, and scheduling/BMS logs) were not collected or accessible for this study.*

In addition, elevated nighttime light variability recorded in one of the single bed rooms during the first monitoring period is likely to have been operational rather than environmental in origin. Periods of room vacancy, cleaning, or preparation between admissions may have contributed to intermittent light use, inflating apparent variability compared with the continuously occupied shared rooms. This limitation highlights the influence of transient operational factors on environmental datasets and reinforces the importance of pairing environmental monitoring with real-time occupancy data in future studies.

This study measured light using photopic lux, which does not account for spectral quality or biological effectiveness. More precise circadian metrics, such as mEDI, were beyond the study's scope but should be considered in future work. Although spectral data were not collected, which prevents calculation of circadian-specific indices such as mEDI or EML, the use of photopic lux provided a practical and validated proxy for evaluating lighting adequacy and rest-phase disruption in an occupied clinical context. Future studies could extend this approach using portable spectroradiometers to quantify spectral composition where feasible. Similarly, sound is reported in measured objective intensity, not subjective experience, and thus does not capture the full psychosocial impact of environmental stimuli.

Although this study included sub-hourly environmental data, detailed clinical activity logs were not available to support full attribution. While noise and light exceedance clusters aligned with known shift overlaps, no timestamped records of staff movements, task types, or patient routines were available for correlation. As a result, operational inference relies on temporal association rather than direct linkage. Future research should include concurrent workflow mapping or timestamped staff logs to enable stronger attribution of environmental patterns to specific operational activities.

Finally, although thresholds were benchmarked against published recommended thresholds, no direct correlation was drawn between environmental conditions and clinical outcomes such as sleep disturbance or delirium incidence. This was a deliberate choice to minimize patient burden, but it limits the ability to determine whether the observed environmental conditions directly caused clinical outcomes such as sleep disturbance or delirium.

## Conclusion

This study demonstrates the critical importance of evaluating environmental performance in PCUs through objective, data-driven methodologies. By employing continuous datalogger monitoring of sound, light, and temperature within a functioning PCU, the research offers empirical evidence of persistent misalignments between current environmental conditions and evidence-based benchmarks for patient well-being. These findings reveal that even well-functioning healthcare environments can unknowingly sustain conditions that have the potential to disrupt sleep, compromise comfort, and impede clinical communication.

The significance of this work lies not only in identifying these environmental shortcomings but in highlighting how they can be quantified, visualized, and ultimately remedied through integrated design and operational responses. The data collected reveal a sensory landscape that fails to fully support the therapeutic goals of palliative care, especially with respect to sound level exceedances, inadequate circadian lighting, and static temperature profiles. Importantly, the study validates the utility of monitoring as a scalable POE tool, offering facility managers and clinical leaders a reliable pathway to assess, benchmark, and improve environmental performance in real time.

This methodology, although applied in a single PCU context and despite the constraints, has broader implications for healthcare design and governance. It provides a scalable, minimally intrusive, and replicable model for conducting rigorous POEs in sensitive clinical settings and embedding environmental quality metrics into standard operational reviews, supporting a shift toward treating environmental data as a core component of patient-centered care while recognizing that broader normalization requires harmonized multisite datasets. The methodology can be adapted to inform design refinements, policy development, and updates to standards such as the Australasian Health Facility Guidelines.

The integration of short-interval noise and light monitoring enabled identification of temporal clusters that closely align with clinical shift overlaps, reinforcing the operational origin of environmental burden in PCUs.

In EoL settings, attention to detail regarding patient comfort is of heightened importance, thus objective measurement and benchmarking are a necessary precursor to optimization of the built environment, which carries both technical and moral significance. The value of this research lies not only in its site-specific findings but also in its demonstration of how the built environment can and should be systematically evaluated as a modifiable contributor to quality palliative care.

*In EoL settings, attention to detail regarding patient comfort is of heightened importance.*

## Implications for Practice

This research has implications for practice in several ways:

- Continuous environmental monitoring offers designers a practical, data-driven method to evaluate whether built environments perform as intended, particularly in relation to sleep, comfort, and sensory experience in high-acuity settings.
- The study highlights the need for architects and engineers to design lighting systems that actively support circadian regulation, using spatial orientation, spectral control, and task-specific fixtures to differentiate between clinical and restorative needs.
- Acoustic exceedances identified in multi-bed rooms reinforce the value of early-stage collaboration between designers and clinicians to mitigate noise through planning, surface materials, and workflow zoning.
- Flat temperature profiles observed across all rooms suggest the need for designers to advocate for HVAC systems that enable diurnal variation and user-responsive control, particularly in spaces where patient thermoregulation is compromised.
- Post-occupancy evaluation using sensor technology should be incorporated as standard practice in healthcare architecture, positioning designers as long-term contributors to performance-based outcomes rather than one-time project deliverers.
- This research supports a shift in the role of the healthcare designer from meeting compliance standards to actively shaping environments that enhance clinical quality, staff wellbeing, and patient dignity at the end of life.

## Highlights

- Continuous monitoring of light, sound, and temperature in a functioning palliative care unit
- Reveals persistent misalignment with sleep-supportive environmental thresholds
- Identifies overlapping sensory stressors that impact patient rest and staff workflow
- Supports design interventions for circadian lighting, acoustic zoning, and thermal variation
- Demonstrates scalable POE method for future healthcare design and evaluation.

## Supplemental Material

sj-docx-1-her-10.1177_19375867261455034 - Supplemental material for Evaluating Environmental Ward Performance: Insights From Acoustic, Lighting, and Temperature Monitoring in a Palliative Care Unit Supplemental material, sj-docx-1-her-10.1177_19375867261455034 for Evaluating Environmental Ward Performance: Insights From Acoustic, Lighting, and Temperature Monitoring in a Palliative Care Unit by Emil E. Jonescu, Benjamin Farrell, Chamil Erik Ramanayaka, Lori Delaney, Edward Litton, Talia J. Uylaki, Gareth Watts, Sadaf Asad, Bella Brigham, Frances Bellemore and Davinia Seah in HERD: Health Environments Research & Design Journal
