# Supplementary material for: Evaluating Environmental Ward Performance: Insights From Acoustic, Lighting, and Temperature Monitoring in a Palliative Care Unit
Source: HERD. 2026 Jun 10;19(4):73–93. doi: 10.1177/19375867261455034 (PMC13624315; doi:10.1177/19375867261455034)
Supplement: Supplementary file 1 [file sj-docx-1-her-10.1177_19375867261455034.docx]

**Appendix Table S1.** Summary of LAeq, Lmax, and Lmin by room type. Deployment 1 (Dec 5–24, 2024) data readings greater than 55 dB(A) in red. Source: Authors, 2025.

| **DAYTIME SOUND LEVELS (7am to 10pm)** | | | |  | |  | | |
| --- | --- | --- | --- | --- | --- | --- | --- | --- |
|  | **Single Bed Room (Room 8)** | |  | | **Four-Bed Room (Room 4)** | | |  |
| **Date** | **LAmax(f)** | **LAeq** | **LAmin** | | **LAmax(f)** | | **LAeq** | **LAmin** |
| Dec_5 | 60.9 | 57.4 | 41 | | 64.6 | | 57.5 | 40.6 |
| Dec_6 | 54.9 | 47.9 | 41.2 | | 66.5 | | 58.5 | 42.8 |
| Dec_7 | 51.8 | 46.6 | 42 | | 66.5 | | 59.6 | 42.9 |
| Dec_8 | 60.5 | 53.7 | 42 | | 63.2 | | 57.5 | 41.5 |
| Dec_9 | 55.9 | 56.1 | 41.5 | | 66.8 | | 59.3 | 42.8 |
| Dec_10 | 58.8 | 56.3 | 41.6 | | 65.6 | | 60.9 | 43.4 |
| Dec_11 | 63.4 | 63.4 | 42.2 | | 65.5 | | 58.7 | 42 |
| Dec_12 | 56.6 | 56 | 41.2 | | 65.5 | | 58.8 | 41.9 |
| Dec_13 | 63.2 | 62 | 39.8 | | 66.6 | | 58.4 | 41.9 |
| Dec_14 | 63 | 61.4 | 39 | | 63.3 | | 57.2 | 41.1 |
| Dec_15 | 61.8 | 61.9 | 40 | | 62.6 | | 55.5 | 40.8 |
| Dec_16 | 68.9 | 64.7 | 40.3 | | 65.2 | | 57.9 | 41.5 |
| Dec_17 | 63.3 | 63.8 | 40.1 | | 65.4 | | 56.5 | 41.8 |
| Dec_18 | 64.3 | 62.5 | 40 | | 57.6 | | 54.6 | 40 |
| Dec_19 | 62.3 | 61.6 | 39.7 | | 58.5 | | 54.2 | 38.7 |
| Dec_20 | 62.6 | 61.9 | 39.1 | | 62.9 | | 54.8 | 39.4 |
| Dec_21 | 62.1 | 60.8 | 39 | | 61.4 | | 54.5 | 39.8 |
| Dec_22 | 59.8 | 59.6 | 39.7 | | 60 | | 51.8 | 38.7 |
| Dec_23 | 60.6 | 60.2 | 38.6 | | 63.5 | | 53.5 | 39.8 |
| Dec_24 | 60.8 | 60.7 | 39.6 | | 61.9 | | 54.6 | 39.7 |
| **Mean** | **60.8** | **60.7** | **40.4** | | **63.7** | | **57.3** | **41.1** |
|  |  |  |  | |  | |  |  |
| **NIGHT SOUND LEVELS (10pm to 7am)** | | |  | |  | |  |  |
|  | **Single Bed Room (Room 8)** | |  | | **Four-Bed Room (Room 4)** | | |  |
| **Date** | **LAmax(f)** | **LAeq** | **LAmin** | | **LAmax(f)** | | **LAeq** | **LAmin** |
| Dec_5to6 | 54 | 50 | 40.2 | | 60.1 | | 56.8 | 40.2 |
| Dec_6to7 | 49.7 | 46 | 40.6 | | 64.5 | | 59.4 | 43.7 |
| Dec_7to8 | 61 | 53.5 | 39.6 | | 61.5 | | 55.8 | 40.8 |
| Dec_8to9 | 58.2 | 51.4 | 40.8 | | 59.5 | | 55.9 | 42.5 |
| Dec_9to10 | 52.8 | 49 | 40.9 | | 62.8 | | 58.1 | 41.9 |
| Dec_10to11 | 54.8 | 62 | 38.3 | | 58.3 | | 57.8 | 38.7 |
| Dec_11to12 | 52.6 | 51.4 | 40.8 | | 63.8 | | 58.9 | 40.3 |
| Dec_12to13 | 53.5 | 57.4 | 41.1 | | 60.5 | | 57.8 | 41.6 |
| Dec_13to14 | 52.7 | 53.8 | 37.3 | | 61.6 | | 56.6 | 39.9 |
| Dec_14to15 | 53.6 | 53.8 | 37.6 | | 58.2 | | 55.3 | 39 |
| Dec_15to16 | 54.3 | 53.7 | 37.5 | | 60.3 | | 54.5 | 40.2 |
| Dec_16to17 | 54.8 | 53.5 | 38.4 | | 59.7 | | 55.5 | 40.1 |
| Dec_17to18 | 57.8 | 52.7 | 37.9 | | 58.5 | | 53 | 39.1 |
| Dec_18to19 | 54.1 | 55.6 | 38.5 | | 55 | | 54.2 | 38.7 |
| Dec_19to20 | 57.6 | 55.5 | 37.5 | | 56.2 | | 52.6 | 38.2 |
| Dec_20to21 | 57.3 | 52.3 | 37.2 | | 56.7 | | 49 | 38.7 |
| Dec_21to22 | 53 | 56.9 | 37.5 | | 56.5 | | 48.3 | 37.4 |
| Dec_22to23 | 54.2 | 51.2 | 37.5 | | 57.6 | | 49.6 | 39.3 |
| Dec_23to34 | 54.8 | 50.8 | 36.6 | | 58.1 | | 53.5 | 37.5 |
| Dec_24to45 | 55.2 | 57.1 | 38.5 | | 57.4 | | 50.6 | 37.5 |
| **Mean** | **54.8** | **54.9** | **38.7** | | **59.3** | | **55.7** | **39.8** |
|  |  |  |  | |  | |  |  |
| **Day/night diff** | **6.0** | **5.8** | **1.7** | | **4.3** | | **1.6** | **1.3** |

**Appendix Table S2:** Summary of LAeq, Lmax, and Lmin by room type. Deployment 2 (Jan 17–Feb 16, 2025) data readings greater than 55 dB(A) in red. Source: Authors, 2025. Source: Authors 2025.

| **DAYTIME SOUND LEVELS (7am to 10pm)** | | | |  |  | | |
| --- | --- | --- | --- | --- | --- | --- | --- |
|  | **Single Bed Room (Room 14)** | |  | **4 Bed Room (Room 13)** | | |  |
| **Date** | **LAmax(f)** | **LAeq** | **LAmin** | **LAmax(f)** | | **LAeq** | **LAmin** |
| Jan_17 | 63.1 | 54.8 | 45.8 | 64.7 | | 55.2 | 48.4 |
| Jan_18 | 62.5 | 54.5 | 44.4 | 63.7 | | 54.3 | 47.9 |
| Jan_19 | 61.7 | 57.1 | 44 | 60 | | 52 | 47.5 |
| Jan_20 | 61.7 | 54.6 | 44.5 | 60.8 | | 52.4 | 47.8 |
| Jan_21 | 62.2 | 57.6 | 45 | 64.3 | | 55.4 | 47.4 |
| Jan_22 | 63.6 | 56.9 | 43.4 | 62.6 | | 53.9 | 47.8 |
| Jan_23 | 62.3 | 56.4 | 42.6 | 62.2 | | 55.2 | 47.2 |
| Jan_24 | 64 | 57.2 | 43.4 | 59.9 | | 52.6 | 47.2 |
| Jan_25 | 59.9 | 53 | 42.3 | 57 | | 51 | 47.1 |
| Jan_26 | 59.9 | 51.8 | 42.1 | 58 | | 52.4 | 47.2 |
| Jan_27 | 61.9 | 55.9 | 43.6 | 59.1 | | 51.9 | 47 |
| Jan_28 | 64.2 | 57.7 | 44.6 | 60.9 | | 53.8 | 47.2 |
| Jan_29 | 66.2 | 58.1 | 43.8 | 60.5 | | 53.1 | 47.2 |
| Jan_30 | 64.7 | 57.3 | 43.5 | 58.1 | | 52 | 46.6 |
| Jan_31 | 63.7 | 56.3 | 43.3 | 57.1 | | 50.5 | 46.7 |
| Feb_1 | 62.2 | 55.7 | 42.9 | 56 | | 51 | 47.1 |
| Feb_2 | 60.3 | 55.9 | 42.2 | 55.5 | | 50.2 | 47.3 |
| Feb_3 | 63.3 | 54.9 | 43 | 61.4 | | 55.5 | 47.3 |
| Feb_4 | 63 | 58.7 | 43.3 | 60.6 | | 54.7 | 47.9 |
| Feb_5 | 64.1 | 55.6 | 43 | 59.8 | | 53.2 | 47.2 |
| Feb_6 | 64.2 | 56.6 | 43.1 | 61.5 | | 54.8 | 46.9 |
| Feb_7 | 63 | 57 | 43.3 | 60.7 | | 54.5 | 47 |
| Feb_8 | 62.9 | 56.4 | 42.9 | 59.2 | | 52.6 | 46.6 |
| Feb_9 | 61 | 53.3 | 42.6 | 57.6 | | 50.7 | 46.6 |
| Feb_10 | 64 | 55 | 43.9 | 67.8 | | 62.7 | 47.9 |
| Feb_11 | 61.4 | 52.8 | 43.4 | 69.7 | | 64.2 | 49 |
| Feb_12 | 63.4 | 55.2 | 44.2 | 67.7 | | 64.2 | 47.3 |
| Feb_13 | 64.3 | 56.6 | 44.1 | 68.4 | | 64.6 | 47.7 |
| Feb_14 | 63.8 | 56.3 | 44 | 68.7 | | 64.1 | 50.2 |
| Feb_15 | 65.3 | 55.8 | 42.8 | 66.4 | | 63.7 | 49.9 |
| Feb_16 | 64.6 | 53.7 | 42.7 | 65.8 | | 64.8 | 49.7 |
| **Mean** | **63.0** | **56.0** | **43.5** | **61.8** | | **58.7** | **47.6** |
|  |  |  |  |  | |  |  |
| **NIGHT SOUND LEVELS (10pm to 7am)** | | |  |  | |  |  |
|  | **Single Bed Room (Room 14)** | |  | **4 Bed Room (Room 13)** | | |  |
| **Date** | **LAmax(f)** | **LAeq** | **LAmin** | **LAmax(f)** | | **LAeq** | **LAmin** |
| Jan_17to18 | 58.7 | 50 | 43.5 | 59.5 | | 53 | 47.6 |
| Jan_18to19 | 59.2 | 52 | 42.6 | 60.5 | | 52.5 | 47 |
| Jan_19to20 | 60.9 | 58.5 | 43.6 | 57 | | 50.2 | 46.9 |
| Jan_20to21 | 57.7 | 54.9 | 43.5 | 58.1 | | 52.6 | 47.5 |
| Jan_21to22 | 58.7 | 58.7 | 43.4 | 58.4 | | 51.4 | 47.3 |
| Jan_22to23 | 56.7 | 50 | 41.5 | 57.3 | | 51.3 | 47.4 |
| Jan_23to24 | 56.9 | 49.7 | 41.6 | 57.1 | | 51 | 47 |
| Jan_24to25 | 58 | 51.8 | 41.5 | 57 | | 50.4 | 46.7 |
| Jan_25to26 | 56.8 | 49.5 | 40.9 | 56.9 | | 50.4 | 46.6 |
| Jan_26to27 | 59.7 | 50.2 | 41.4 | 56.6 | | 51.8 | 46.6 |
| Jan_27to28 | 62.5 | 53.4 | 42.2 | 58.4 | | 52.5 | 46.8 |
| Jan_28to29 | 58.6 | 54.6 | 42.4 | 59.4 | | 52.2 | 46.6 |
| Jan_29to30 | 59.9 | 54.6 | 41.8 | 55 | | 51.1 | 46.8 |
| Jan_30to31 | 57 | 50.6 | 42.6 | 45.3 | | 49 | 46.1 |
| Jan31toFeb1 | 56.9 | 50 | 41.5 | 56.2 | | 53.6 | 46.8 |
| Feb_1to2 | 56.9 | 52.5 | 41.2 | 54.8 | | 50.6 | 46.7 |
| Feb_2to3 | 57.5 | 50.3 | 42.1 | 55 | | 50.3 | 46.9 |
| Feb_3to4 | 59.2 | 49.6 | 41.3 | 62.9 | | 55.9 | 47.5 |
| Feb_4to5 | 61.9 | 54.4 | 42.1 | 56.6 | | 52.1 | 46.8 |
| Feb_5to6 | 61.7 | 53.7 | 41.1 | 56.1 | | 50.2 | 46.5 |
| Feb_6to7 | 58.9 | 51.3 | 41.8 | 56.6 | | 51.4 | 46.1 |
| Feb_7to8 | 58.4 | 51.4 | 40.9 | 56.8 | | 51.2 | 46.4 |
| Feb_8to9 | 58.1 | 50.5 | 41.9 | 55.8 | | 49.7 | 46.3 |
| Feb_9to10 | 60.3 | 52 | 42.6 | 56.7 | | 51 | 46.5 |
| Feb_10to11 | 62.2 | 54.6 | 42.6 | 69.6 | | 65.6 | 49.7 |
| Feb_11to12 | 59.4 | 52.5 | 42.7 | 59.7 | | 60.4 | 48.2 |
| Feb_12to13 | 59 | 51.9 | 42.3 | 62.5 | | 61.9 | 46.8 |
| Feb_13to14 | 57.8 | 50.7 | 43.1 | 62.9 | | 61 | 47.2 |
| Feb_14to15 | 57.2 | 51.1 | 43.7 | 61.9 | | 61.3 | 47.9 |
| Feb_15to16 | 61.9 | 50.5 | 41.3 | 63 | | 61.6 | 49.2 |
| Feb_16to17 | 64.9 | 54.6 | 42.4 | 62.6 | | 60 | 49.3 |
| **Mean** | **59.1** | **53.1** | **42.2** | **58.3** | | **56.8** | **47.2.8** |
|  |  |  |  |  | |  |  |
| **Day/night diff** | **3.8** | **3.0** | **1.7** | **3.5** | | **1.9** | **0.5** |
